# Scanning the horizon: deep mutational scanning approaches in virology

**DOI:** 10.1128/jvi.01784-25

**Published:** 2026-06-10

**Authors:** Jack Dorman, William Bakhache, Patrick T. Dolan

**Affiliations:** 1 Quantitative Virology and Evolution Unit, Laboratory of Viral Diseases, Division of Intramural Research, National Institutes of Allergy and Infectious Diseases, National Institutes of Health 2511, Bethesda, Maryland, USA; 2 Department of Biology, Johns Hopkins University 228291, Baltimore, Maryland, USA; 3 Université de Strasbourg, CNRS UPR9022, Institut de Biologie Moléculaire et Cellulaire, Strasbourg, France; Indiana University Bloomington, Bloomington, Indiana, USA

**Keywords:** deep mutational scanning, virus evolution, mutation

## Abstract

Studying the effects of mutations is central to virology. Deep mutational scanning (DMS) is a technique that couples high-throughput mutagenesis with deep sequencing to measure the effects of mutations in pooled assays. When adapted to viruses, DMS accelerates comprehensive measurement of mutational effects across viral genomes, mapping evolutionary constraints on viral proteins. Recently, DMS has been applied to a wider variety of problems in virology and viral evolution, quantifying the effects of mutation across host species, tissue environments, and immunological pressures. Since DMS was first applied to virology, synthetic biology has transformed the engineering of mutational libraries, opening new questions to exploration. With the technology now matured, DMS is poised to transform our understanding of viral evolution in new, exciting ways. This review will synthesize recent technological and conceptual advances in DMS methods being applied to virology, the insights it is yielding, and the opportunities for future studies.

## INTRODUCTION

**Fig 1:**
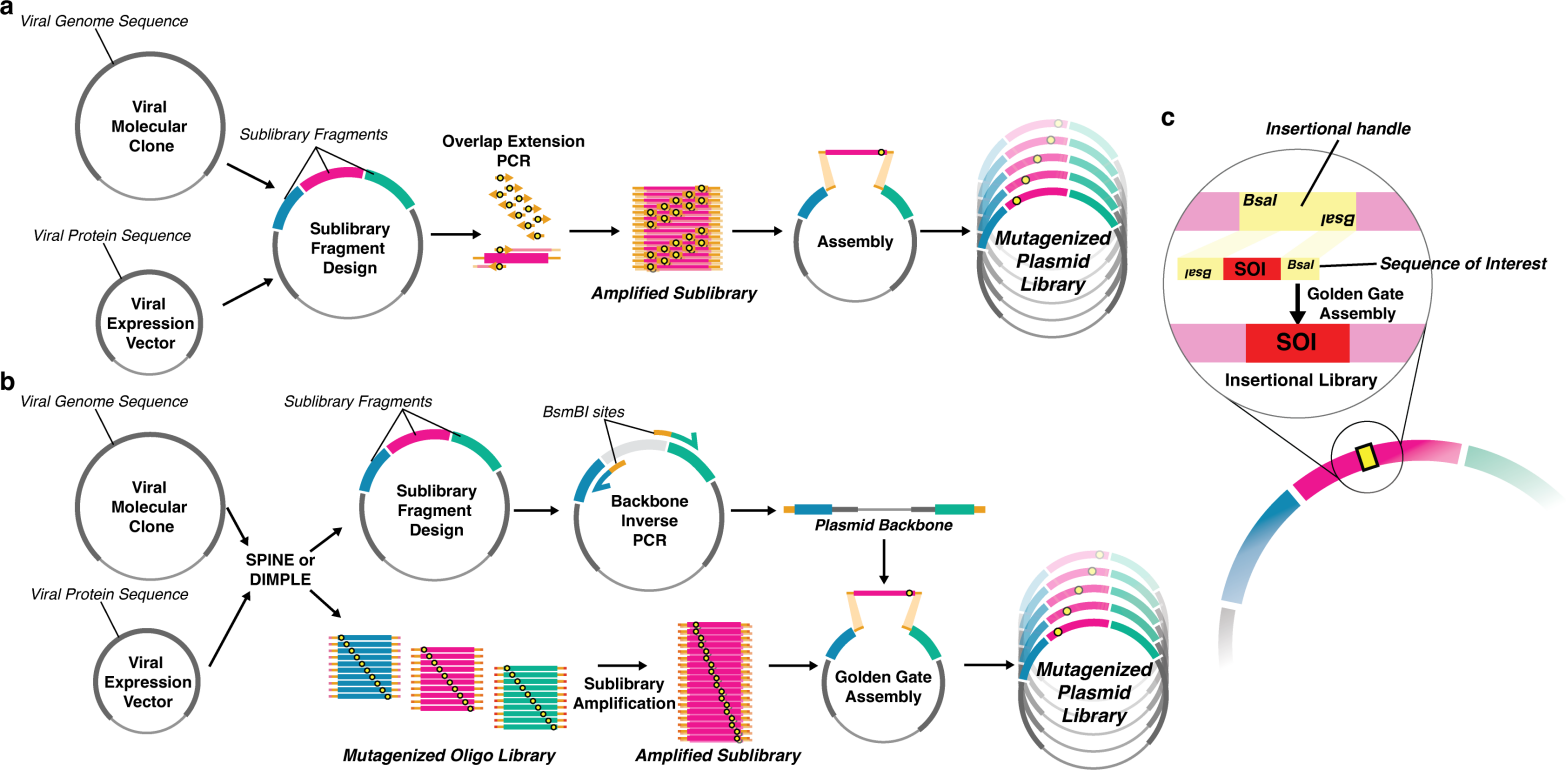
Common approaches to generate mutational scanning libraries in viral coding sequences. (**a**) Degenerate primer approach. Primers are designed with degenerate codons at each target position in the coding sequence. The workflow proceeds in the following stages: (i) PCR amplification of the target region from a template plasmid; (ii) separate mutagenic PCR reactions using forward and reverse degenerate primers; and (iii) a joining PCR to assemble the full-length mutagenized product. The final PCR product is then ligated into the target plasmid (infectious clone or expression vector) using any preferred cloning method. (**b**) Oligopool approach. The complete mutant library is designed across defined sub-libraries using SPINE or DIMPLE organized by genomic position into manageable subsets (in terms of the number of engineered variants). First, an inverse PCR is performed on the plasmid backbone, either an infectious molecular clone or expression vector, depending on the experimental platform. The inverse PCR amplifies everything, except the target region, to be mutagenized. Second, the inverse PCR product is assembled via BsmBI Golden Gate cloning with the corresponding PCR-amplified mutagenized oligopools. This generates a mutagenized plasmid sub-library. These sub-libraries can be pooled into a larger experimental library, or data from each sub-library can be combined after the experiment for joint analysis depending on the scale and resolution required. (**c**) Design of the insertional handle. This represents one type of mutation that can be introduced through the oligopool approach. The genetic handle contains outward-facing Type II BsaI restriction sites. These sites allow for the rapid insertion of any sequence of interest (SOI) into the plasmid.

The development of deep mutational scanning (DMS) approaches has led to a fundamental shift in our ability to connect viral genotype and phenotype (1) and accelerated our understanding of viral evolution and protein function. For the purposes of this review, DMS refers to a suite of approaches that use synthetic biology to engineer diversity into a given target protein, followed by assessment of the phenotypic effects of each mutation by sequencing, either over successive passages or before and after selection (Fig. 1a). The methods used to generate libraries of variants, the types of mutations able to be explored, and the systems and biological questions to which DMS is being applied is ever-expanding, and we hope to provide a broad overview of the current advances and opportunities in the field, along with practical experimental and analytical factors to consider when designing such studies.

DMS approaches are especially amenable to questions in virology. On one hand, viruses are relatively simple genetic systems often able to be produced from a single plasmid or several plasmids, generating a fully infectious virus of a known genotype. Many aspects of the viral lifecycle are governed by single gene products; entry is often mediated by a single fusion protein, protein complex, or set of capsid proteins; replication by a single polymerase. This enables experimenters to interrogate multiple functions more comprehensively through mutagenesis than is possible for more complex cellular processes. Conversely, viral proteomes are complex. Viral proteins are often multifunctional, form complexes with both viral and host proteins, and respond to environmental and cellular signals to coordinate their functions throughout the viral lifecycle. Although DMS has been primarily used to measure the tolerance of viral proteins to mutation, as DMS approaches and experimental designs expand, they allow more precise dissection of the functional and structural constraints of viral proteomes across environmental and cellular conditions or at specific stages of viral lifecycles.

DMS is just one approach to experimental evolution, complementing other methods and approaches. Conventional ‘passage-and-sequence’ experimental evolution is limited in its ability to comprehensively and quantitatively survey selective pressures on viral genomes accessible through mutation or which exist in the standing variation of the initial population. However, with sufficient passages, populations will identify complex evolutionary solutions often involving multiple mutations (2–5). By contrast, DMS is primarily focused on the effects of single mutations, allowing the experimenter to engineer variation into a population, and then assess the mutational fitness effects of each mutation after passage in specific selective conditions. This comprehensive, parallel approach reveals the entire local selective landscape available to a given parental genotype, allowing us to compare the effects of all possible mutational steps simultaneously. Compared to longer-term passaging, this reveals a very high-resolution picture of a very limited region of ‘sequence space.’

As the growing literature demonstrates, DMS is highly adaptable to problems in virology at any scale, such as dissecting the role of specific substitutions in a defined active site or antibody epitope, an entire protein domain, or across an entire viral genome. The insights gained are similarly diverse, defining the evolutionary constraints exerted by physical, biological, and genetic factors or providing specific mechanistic insights into protein structures or enzyme functions. Here, we attempt to anticipate where the field is headed and the new experimental paradigms that will further expand our understanding of basic and translational aspects of viral evolution.

## DEEP MUTATIONAL SCANNING APPROACHES

### Technological development

The field of DMS has evolved rapidly since the early 2000’s, when the advent of high-throughput sequencing technologies made it possible to identify engineered variants in parallel through large sequencing screens. In the last decade, these technologies have matured, enabled by advances in synthetic biology tools that have given researchers more precise control over mutational library diversity and the types of mutations that can be assessed in DMS assays.

Although the approach we commonly refer to as DMS was developed in 2011 (1, 6, 7), it builds on several earlier technologies that took advantage of high-throughput sequencing to perform pooled screens of engineered mutant libraries. These early technologies included transposon mutagenesis to explore insertional tolerance and functional constraints in proteins (8–12) and chemical mutagenesis using mutagenic bases (13) or error-prone DNA polymerases to introduce diversity into amplicon pools (14, 15). These early approaches gave way to more refined approaches that enable precise control over library diversity.

The first commonly used approach for generating mutational libraries employs pools of primers tiled across the target protein, each encoding degenerate codons (Fig. 1a). Pooled amplification of the target region introduces codon substitutions across the target sequence (16, 17). Primers targeting each site in the coding region to be mutagenized are pooled in equimolar ratios in the amplification reaction. Methods to apply this approach to your viral system can be found in the *CodonTilingPrimers* GitHub page (18).

More recently, library design using synthetic oligonucleotide pools (‘oligopools’) has become the predominant methodology for DMS in viral systems (19–23), driven by advances in high-throughput oligopool synthesis from companies like Twist Bioscience or GenScript. These technologies enable researchers to directly synthesize sequences with designed mutations of interest and clone them into the target plasmid (Fig. 1b). Computational tools, such as Saturated Programmable INsertion Engineering (SPINE) and Deep Indel Missense Programmable Library Engineering (DIMPLE) (24–28), have further streamlined this process by automating the design. Users need only provide their template open reading frame (ORF) sequence and specify the desired mutation types, and these pipelines will produce both the primers to amplify the plasmid backbone for each sublibrary and the cognate oligopool sequences.

These two primary approaches, pooled PCR with degenerate primers or library generation from synthetic oligopools, have distinct advantages and disadvantages that should be considered prior to library generation. The degenerate primer approach offers a balanced combination of cost-effectiveness and experimental design flexibility. It enables generation of mutational scanning libraries with near-complete amino acid substitution coverage at lower cost than synthetic oligopool-based methods. However, this method has notable limitations: it cannot efficiently generate insertions and deletions and becomes increasingly tedious for large-scale scans covering substantial portions of viral genomes. The pooled nature leads to dropout of some variants while producing some amplicons with multiple codon substitutions. Possible confounding epistatic effects complicate phenotypic interpretation and require specific models to account for these interactions (29, 30).

By contrast, oligopool-based strategies offer more robust and versatile design options. They provide precise control over codon usage to maximize multi-nucleotide substitutions, which helps reduce variant miscalling due to sequencing errors. They also produce more complete mutational libraries with more consistent and uniform variant frequencies. Finally, sublibrary architecture provides unique experimental flexibility: researchers can pool all sublibraries for comprehensive genome-wide coverage or selectively use specific sublibraries for targeted studies without regenerating the entire library. The primary disadvantages of this approach are the cost of synthetic oligopools, which scales with library complexity, and the inherent synthesis error rate of oligopools (approximately one error per 3,000 nucleotides) (31). While synthetic oligopools are more expensive upfront than degenerate primers, the reduced hands-on time, improved library quality, and flexibility to explore various mutation types may offset costs for comprehensive or large-scale studies. These advantages have led most researchers to transition to oligopool-based strategies for library design.

### Biosafety and non-infectious systems

Of course, an important consideration for viral DMS screens, particularly highly pathogenic viruses or those with pandemic potential, is biosafety and potential gain-of-function experiments. These concerns have spurred the development of many systems to study viral proteins from such pathogens in non-infectious systems. These include non-infectious reporters, such as pseudotyped virus particles (using vesicular stomatitis virus (VSV) [32], lentivirus [33]), bacteriophage (34, 35), or yeast-display methods (36, 37). These tools have enabled screens of individual proteins from high-consequence pathogens at lower biosafety levels, including Nipah virus (38, 39), rabies virus (32), and others.

These non-infectious and pseudotyped models have been especially productive for studying the structural proteins of enveloped viruses, such as HA and NA function in influenza, or spike proteins in coronaviruses, where expression and presentation of amenable transmembrane proteins or domains are possible. However, non-enveloped viruses, such as picornaviruses, are not amenable to such viral pseudotyping or surface display strategies. In these cases, studies have been primarily performed in the context of infectious virus. Although these studies are more challenging technically and limited to work at higher biosafety levels and with fewer selective pressures, the ability to examine mutational effects in the context of viral infection has allowed the study of functional constraints in both structural and non-structural proteins. In addition, many families of pathogenic viruses have so-called ‘prototypes,’ attenuated viruses or less pathogenic members, that enable measurement of constraint in fully infectious systems (40).

Further mitigating potential risks, previous DMS work using infectious systems demonstrates that the vast majority of mutations do not enhance viral fitness, and those that do are produced in very small numbers in pooled assays. Moreover, even if a mutation exhibits increased fitness *in vitro*, in most cases, fitness in cells may not predict the emergence of a specific mutant in the more complex selective environments in cells or in host populations. Still, rigorous biosafety considerations remain essential for evaluating every infectious DMS screen.

### Analysis of variant effects for DMS experiments

At a basic level, the estimation of variant effect, or fitness effect, from DMS experiments is simply the fold enrichment of mutations after selection compared to the unselected input population in ‘two-sample’ experimental designs or the rate of increase or decrease in frequency in passage experiments estimated by regression analysis. Mutations, which increase in frequency after selection, are more fit, while those that decrease or are removed from the population are deleterious or lethal. However, the complexity of the libraries, experimental designs, and sources of experimental noise necessitate more complex statistical models to estimate variant effects. As experimental approaches have advanced, and library complexity has increased, so too have approaches to analyze DMS data sets. A comprehensive review of the computational approaches and software for variant counting and variant effect estimation was recently published (41). However, for virus-focused research, several DMS studies also rely on custom scripts for variant effect calculation. Each tool differs in its statistical approach (frequentist vs. Bayesian) and the types of experimental designs they can accommodate (e.g., time series vs. two-sample). They also have unique advantages and disadvantages in terms of computational efficiency and the types of input and output formats, including various approaches for data visualization (summarized in Table 1). It is critical to consider the analysis tools that will be employed downstream of your study prior to library design, as strategic choices at this early stage will improve data quality and interpretation. One important example is the inclusion of synonymous or wild-type variants in the library, which can be used by many software packages to normalize the variant effects observed against a neutral reference.

**TABLE 1:** Summary of analysis method for calculating mutational effects and visualizing data

Tool	Experimental designs supported	Key statistical approach	Typical inputs	Output/ interpretation	Language/ interface	Notable strengths	Limitations
*Enrich* (7)	Two-population (input vs. selected)	Log_2_ enrichment ratio	FASTQ reads	Variant enrichment scores	Python (CLI)	First dedicated DMS tool; simple and widely used	Python 2 only; limited modeling; no time series
*Enrich2* (42)	Two-population and time-series	Log ratios + linear regression; mixed-effects modeling for replicates	FASTQ, count tables, barcodes	Variant scores with replicate integration	Python (CLI + GUI)	Handles replicates and time series; good diagnostics	Older software stack
*dms_tools2* (43)	Two-population	Log enrichment → amino-acid preference; optional Bayesian inference	FASTQ or codon count tables	Site-specific amino-acid preferences	Python (module/notebooks)	Popular for viral DMS data sets; integrates structural analysis	Focused on site preferences rather than general fitness
*DiMSum* (44)	Two-population	Error-aware enrichment modeling with weighted mean across replicates	FASTQ or variant counts	Variant fitness estimates + uncertainty	R/command line	Strong error model; diagnostic HTML reports	Primarily two-population designs
*Mutscan* (45)	Two-population	Flexible statistical models (user-specified fixed-effects models)	FASTQ reads	Enrichment statistics and tests	R	End-to-end pipeline; flexible modeling	Less widely adopted
*Rosace and Rosace-AA* (46)	Time-series or growth assays	Hierarchical model with mean-variance shrinkage across positions	Count tables	Variant fitness with improved FDR control	R/Python tools	Incorporates positional information for robustness	Newer tool; fewer pipelines
*popDMS* (47)	Time-series	Bayesian population-genetic model	Variant counts	Selection coefficients	Python	Models evolutionary dynamics explicitly	Requires more complex data
*ACIDES* (48)	Time-series	Maximum likelihood estimation of selection coefficients	Variant counts	Fitness/selection estimates	Python	Designed for time-course selection experiments	Less visualization support
*Fit-Seq2.0* (49)	Time-series	Population genetics-based inference	Variant frequency trajectories	Selection coefficients	Python	Good for dynamic fitness estimation	Requires multi-timepoint experiments
*TileSeqMave* (50, 51)	Time-series and tile-based sequencing	Regression-based scoring	Tiled sequencing counts	Variant effect scores	Python	Optimized for tiled sequencing experiments	Limited built-in visualization

DMS data sets are typically visualized using several standard approaches. Initially, scatter plots are common for assessing replicate reproducibility and overall data quality. Static or interactive heatmaps provide a comprehensive view of all variant scores across the mutagenized region, while sequence logo plots (52) intuitively reveal site-specific amino acid preferences. To summarize positional tolerance, per-site effects (derived from mean or median scores) are displayed using line plots. Score distributions are frequently examined using histograms, density plots, or boxplots, with statistical comparisons (e.g., Wilcoxon test) , performed between experimental conditions or structural elements. Finally, mapping per-site variant scores onto three-dimensional (3D) viral protein structures provides spatial context for mutational effects.

### Expanding the mutational repertoire of DMS

Although conventional DMS approaches were focused on the comprehensive assessment of amino acid substitutions, DMS approaches have been expanded to examine the fitness effects of insertions and deletions (19–21, 25, 53) and mutations in non-coding regions of viral genomes (10, 54). This has been accelerated by advances in oligonucleotide library synthesis and library design, which allow the insertion or deletion of specified sequences, opening new opportunities to explore the roles of insertions and deletions of different lengths and biochemical characteristics. The screening of engineered insertions and deletions is reminiscent of the earliest Mu phage-generated insertional screens assessing insertional tolerance, although these new approaches now allow more precise control and overcome the bias associated with transposon insertion to enable complete saturation of target sites within the library (24). One notable innovation, which expands the utility of insertional scanning, is the engineering of a short sequence known as an “insertional handle” at specific sites along a target protein (24). This handle, which encodes outward-facing Class IIS restriction enzyme sites (55) (commonly used in “scarless” Golden Gate cloning [56]), allows insertion of any sequence of interest (or a pool of sequences) into each engineered site (Fig. 1c). Once engineered into each site of the target ORF in a plasmid, digestion of the plasmid pool by the RE allows the same plasmid library to be screened for tolerance to insertion of any sequence of interest. This makes such scans incredibly cost-efficient by avoiding the generation of a new oligopool for each insertion of interest. Although originally designed for the screening of domain insertions in so-called “deep domain scanning” studies of cellular channel proteins (24), we recently adapted it to screen sites in viral proteomes, which tolerate the insertion of affinity tags (e.g., ALFA-tag [57]) or small reporters (e.g., NanoLuc [58]) in an unbiased fashion (20, 59). Performing these screens in the context of replication-competent viral infectious clones and replicons ensures that the tolerated insertions do not interfere with basic functions of the target proteins, making them valuable reagents for virological research (59).

## INSIGHTS ACROSS BIOLOGICAL SCALE

Since its early applications, DMS has been applied to a wide range of viruses, with a predominant focus on the receptor binding proteins of RNA viruses (Table 2). A comprehensive summary of all DMS studies is beyond the scope of this review. We aim, instead, to outline some successful conceptual frameworks employed across different viral systems and levels of biological complexity to help researchers understand how to apply DMS to their own questions.

**TABLE 2:** Summary of eukaryotic viruses with at least a single peer-reviewed publication describing a DMS screen

Species	Genus	Gene(s)	Citation(s)
Sindbis	Alphavirus	nsP3 opal stop codon	60
Mayaro	Alphavirus	E1, E2, E3, 6K	40
Chikungunya	Alphavirus	E1, E2, E3, 6K	61, 62
Lassa	Arenavirus	GPC	63
SARS-CoV-2	Coronavirus	Spike (full or RBD) ×20, 3CL	23, 33, 36, 37, 64–79
Zika	Flavivirus	E ×2, NS2B, NS3	67, 78, 79
West Nile	Flavivirus	E	22
Hepatitis C	Hepacivirus	NS5A ×2	80, 81
HIV	Lentivirus	Env ×4, Tat, Rev, Gag, PR	82 – 88
Rabies	Lyssavirus	G	32
Hepatitis B	Orthohepadnavirus	Rep, Core	89
Influenza A	Orthomyxovirus	HA ×13, NA ×3, NP ×2, M1 ×2, PB1 ×2, PA ×2, PB2, NEP, NS	14–16, 30, 90–115
Nipah	Paramyxovirus	RBP, F	38, 39
AAV	Parvovirus	VP1, VP2, VP3, Rep	19, 53, 116
Coxsackievirus	Picornavirus	Full polyprotein	21, 117
Enterovirus	Picornavirus	Full polyprotein	20

### Dissecting intrinsic molecular constraints

**Fig 2:**
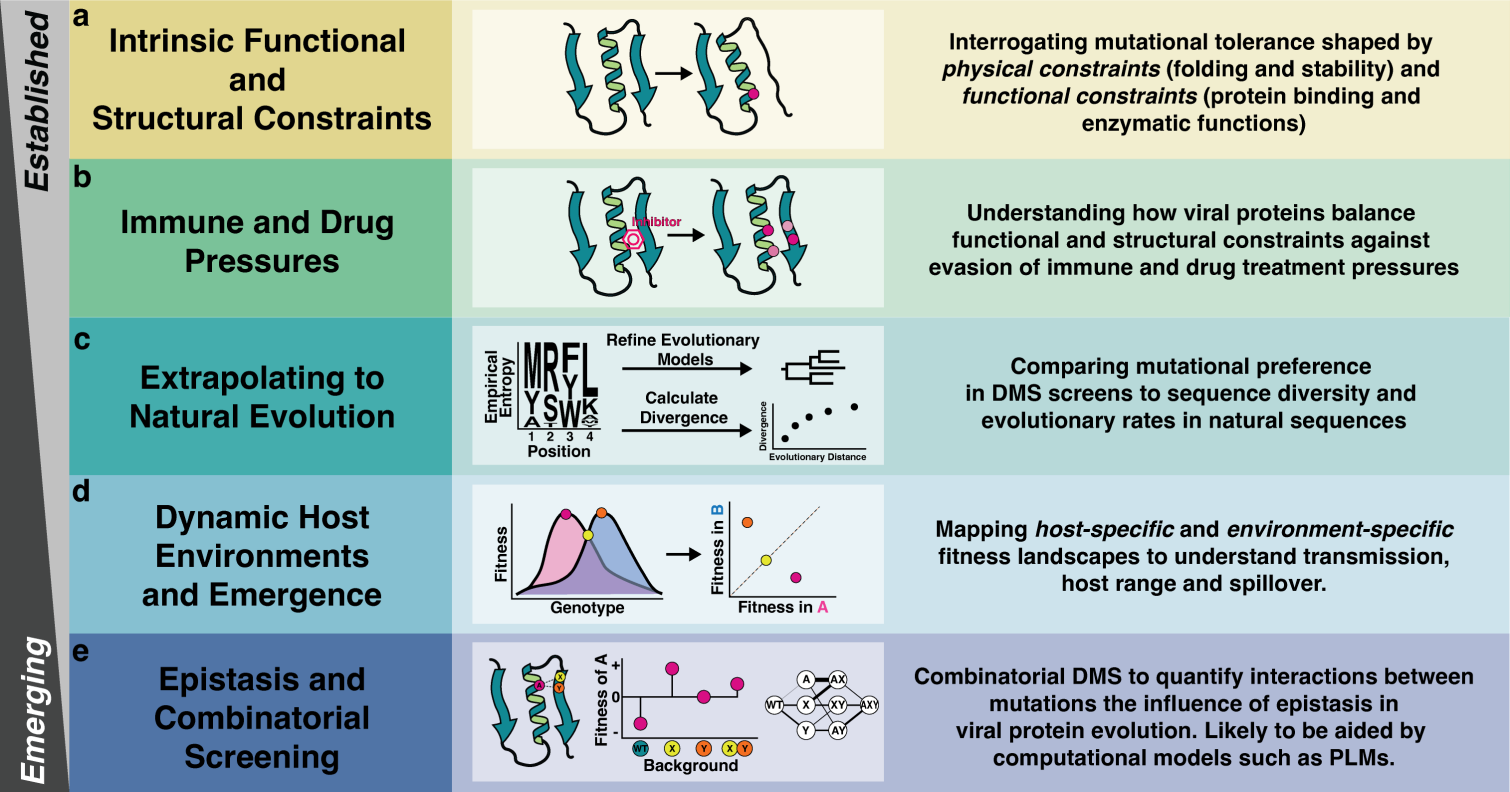
Applications of deep mutational scanning (DMS). DMS has been applied to an increasingly complex set of questions, which we broadly categorize as established or emerging fields of research based on the volume of current literature. (**a**) The core finding of a DMS screen is the measurement of fundamental constraints acting on viral replication driven by the inherent properties of the virus. (**b**) Further studies have incorporated extrinsic selective pressures layered on top of intrinsic viral functions. These pressures are often relevant to medical countermeasures, such as antivirals or antibodies. (**c**) Genomic surveillance and phylogenetic analysis provide essential context for experimentally measured constraints. Because of the complexity of natural evolution, explaining DMS results through the lens of natural viral evolution is challenging, but a growing body of work demonstrates its feasibility. (**d**) The next key challenge for DMS is understanding constraints as they are balanced across a more representative array of environments. Complex host environments, including *in vivo* and organoid models, are often too technically difficult to screen directly. In addition, there is a need for new analytical frameworks to interpret DMS results performed in more complex environments. (**e**) The sheer number of variants required to systematically screen for interactions between mutations (epistasis) remains a challenge, though work in this area is accumulating. By integrating data across all these levels of measurement, a more holistic understanding of viral biology and evolution will emerge.

Fundamentally, DMS interrogates the tolerance of viral proteins to mutation. The tolerance to mutation is shaped by physical constraints, such as the folding and stability of viral proteins, as well as biological or functional constraints, such as the ability to bind viral or host proteins, or perform enzymatic functions (Fig. 2a). These intrinsic constraints are examined to some extent in all DMS studies on viral proteins, informing our understanding of viral protein structure and function.

As an example, several DMS screens in picornaviruses have been recently published (20, 21, 117–120). These viruses include clinically important pathogens, such as enterovirus (EV)-D68, coxsackievirus (CV)-B3 and EV-A71, and poliovirus. Because of their small genomes and simple molecular biology, that is, a 7 kb positive-sense genome encoding a single open reading frame, it has been possible to interrogate the effects of mutation across the entire viral coding region and quantify the evolutionary constraints on both structural and non-structural proteins. Notably, picornaviruses constitute an important evolutionary predecessor to many modern RNA viruses, contributing structural and enzymatic features that are shared among all modern RNA viruses, including the jelly-roll fold and 3C protease (121). Evolutionary constraints acting on these deeply conserved features of the viral genome are likely to inform on constraints across the many viruses that share these same features.

Viruses with compact, information-dense genomes often balance intrinsic constraints across overlapping coding regions. While this can present a technical challenge, it also presents an opportunity to characterize how genome organization itself can exert intrinsic constraints on virus fitness and evolution. Hepatitis B virus (HBV), which expresses multiple overlapping ORFs within the polymerase gene, presents a case to measure the influence of these overlapping constraints. In a recent study (89), variants of the HBV genome were generated that separated the fitness of the polymerase from the overlapping proteins, revealing novel regulatory elements and protein motifs. The HBV polymerase model presents an extreme example of overlapping genomic constraints and demonstrates the value of exploring entangled genes independently.

### Mapping escape from immune pressures

In the case of viral pathogens, the intrinsic constraints that maintain the form and function of viral proteins are balanced against extrinsic selective pressures, including the dynamics of the immune system, shaping their long-term viral evolution (Fig. 2b). The comprehensive nature of DMS has allowed the systematic disentangling of these opposing selective pressures, revealing important differences across pathogens and allowing us to anticipate mutations that are most likely to evade immune recognition, particularly by the adaptive immune system.

The most notable use of DMS to understand immune dynamics was during the COVID-19 pandemic, where DMS studies focused on the RBD and fusion peptide of SARS-CoV-2 spike protein to understand and anticipate mutations that alter viral protein function (36, 37) and evade antibody immunity (64, 122, 123). More recent studies in pseudotyped systems have allowed interrogation of how the entire spike protein navigates these opposing pressures, enabling prediction of successful lineages (33, 65).

Pathways of escape are often more complex than mediating direct interactions between virus and antibody. For example, DMS of the Env glycoprotein of HIV in the presence of a series of broadly neutralizing monoclonal antibodies demonstrated a diverse set of escape mechanisms (82, 124). Many escape mutations were at sites of contact with an antibody but, notably, several were distant from identified epitopes. These screens were highly concordant with escape mutants observed over the course of clinical infections, reinforcing the practical, actionable information that DMS screens can provide in terms of optimizing therapeutics to anticipate resistance.

### Overcoming extrinsic pressure from small molecule inhibitors

In addition to natural extrinsic selective pressures, viruses navigate the selective landscape that we shape with small molecule inhibitors and therapeutic antibodies, often rapidly evolving resistance (Fig. 2b). Chronic viruses, like HIV, readily evolve resistance to individual inhibitors; however, combination therapies have been remarkably effective at controlling HIV infection. Characterizing pathways of escape for these countermeasures with DMS can identify effective combinations that reduce the potential for resistance. Similarly, there is growing work to utilize *in vitro* pathways of resistance to monoclonal Abs in order to optimize treatment strategies (66, 123).

Single mutations that confer resistance often exhibit sharp functional trade-offs. DMS of single amino acid substitutions systematically demonstrates how resistance mutations are poorly tolerated in the absence of selective pressures from inhibitors. Interrogation of the polymerase of hepatitis C virus by DMS in the presence of different levels of an inhibitor revealed that several substitutions, which were highly deleterious in the absence of the inhibitor, became significantly beneficial with increasing doses of the antiviral (80). Similar results have been obtained in studies of EV-A71 (119), suggesting a more global phenomena.

The fitness defects of resistant mutations are often compensated by secondary mutations, which in chronic viruses readily emerge over the course of infection, leading to complex evolutionary pathways underlying resistance. For example, a combinatorial DMS of resistance-associated mutations in the HIV protease in the presence of an inhibitor revealed the complex interactions between mutations, called epistasis, shaping the landscape of escape (83). Multiple distinct combinations of mutations increased protease resistance while compensating for some of the well-characterized reduction in viral replicative fitness. These higher-order genetic interactions compound the challenge of resistance, as they decrease the pressure for a population to revert in the absence of treatment, leading to sustained transmission of resistant variants.

With greater insight into pathways of escape, we can pursue the next clear imperative: anticipating and preventing resistance. The feasibility of this was demonstrated in recent work pairing DMS with crystallographic fragment screening of the NS2B-NS3 genes of Zika virus in order to generate and refine small-molecule antivirals (67). Fragment screening yielded several candidate chemical scaffolds that are plausible inhibitors, and the added context of the DMS results allowed researchers to prioritize those molecules that interacted with highly constrained viral residues. The combined data sets thereby allowed the identification of candidate antivirals refractory to the evolution of resistance. The use of DMS data to design effective countermeasures is a powerful concept that should be widely adopted across viral systems.

### Identifying molecular determinants of host tropism

Viruses often exhibit broad host range or tissue tropism, with important implications for transmission, spillover potential, and pathogenesis. As an extreme example, arthropod-borne viruses, like flaviviruses, make such transitions as an obligatory part of their transmission cycle, balancing fitness between an arthropod vector and vertebrate hosts. These viruses navigate selective landscapes in hosts separated by hundreds of millions of years of evolution, providing a unique opportunity to study how viruses evolve in such dynamic environments. Our recent DMS studies in the envelope protein of West Nile virus examined how fitness changes across cell lines derived from humans, mosquitoes, and birds (the definitive vertebrate host of many flaviviruses) (22). The three-way comparison across environments revealed novel hotspots where constraint differs, particularly in avian environments. Defining the molecular niche that separates definitive vertebrate hosts and vectors from incompetent hosts remains an open and compelling avenue of research which DMS can assist.

Changes in host tropism are not only a feature of pathogenesis but also of medical interventions, such as gene therapy. AAV vectors have been the target of multiple DMS screens in an attempt to optimize such applications. These screens have focused predominantly on the capsid proteins given their essential role in the determination of host tropism (19, 53). The results of these screens include numerous variant AAV capsids with altered tissue tropism, production efficiencies, and/or stability. Furthermore, they have served as training data for machine learning algorithms to enhance the feasibility of AAV engineering. In addition to applied aspects, DMS screens of the AAV capsid have yielded valuable basic biological insights, such as alternative ORFs and functional motifs of capsid proteins.

### Leveraging DMS for outbreak response

The combination of selective pressures across intrinsic, extrinsic, and environmental scales ultimately drives evolutionary change at the population level. Translating this evolutionary framework into real-time outbreak response established DMS as a critical tool during the COVID-19 pandemic. DMS data sets were generated on SARS-CoV-2 sequences within 6 months of the first published sequence (36), providing early indicators of adaptive pathways to human ACE2 binding. Paired with the abundance of genomic surveillance in the early years of the pandemic, researchers had the unprecedented opportunity to assess how well DMS-derived fitness effects capture the evolutionary dynamics of natural populations. Overall, DMS was highly effective at anticipating the emergence of SARS-CoV-2 variants, even factoring in complex immunological environments (37, 65, 125). Researchers could also capture the nuanced epistatic interactions between Spike protein mutations, particularly in the receptor binding domain.

Given the diversity of viral pathogens that exist in cryptic transmission cycles and which have the potential to emerge explosively as SARS-CoV-2 did, generating evolutionary insights before spillover is essential. The effectiveness of DMS in understanding the COVID-19 pandemic has created the impetus to expand coverage to emerging or re-emerging viral pathogens in the hopes of elucidating factors driving spillover and improving countermeasures for these viruses. Such work has already been done on the glycoproteins of Nipah, Lassa, and rabies viruses, all emerging or re-emerging zoonotic viruses (32, 38, 39, 63). These screens further refine understanding of antibody recognition and provide opportunities to improve the efficacy of mAbs to these pathogens. Finally, as we start generating DMS data sets across a viral genus, this will define genetically constrained regions for the design of pan-genus antivirals and vaccine antigens, creating a robust therapeutic arsenal ready for future emerging pathogens.

## CONCEPTUAL CHALLENGES

### Extrapolation of experimental constraint

The ability to extrapolate DMS data to natural evolution of viruses is key to its utility (Fig. 2c). DMS studies are performed in controlled laboratory environments with tight control over population sizes, measuring fitness based on a limited number of phenotypes. This leads to key questions for the field. Are the measurements of fitness generated in a lab, often in cell culture, relevant to natural evolution either within or between hosts? Can data from one virus be applied to related viruses? And what are the limits at which DMS data are no longer predictive of constraint in other environments or related viruses?

The difficulty in translating *in vitro* fitness to selection *in vivo* was nicely demonstrated in a DMS of the E3-E2-6K-E1 envelope proteins of Mayaro virus, an alphavirus (40). Screening in cell culture on monkey- and various mosquito-derived cell lines identified substitutions in the structural protein, E2, with distinct host preferences. However, efforts to screen the libraries in whole mosquitoes and mice yielded unclear signatures of selection and, as a result, the authors resorted to *in vivo* validation of individual mutants identified in their *in vitro* screen, ultimately confirming the observed host-specific fitness trade-offs. This example illustrates the influence of anatomical bottlenecks and diverse selective pressures operating within whole organisms, which obscure the fitness effects of substitutions in mutational libraries. However, some viral systems and host contexts have yielded better results, likely reflecting both differences in experimental design, library complexity, and aspects of the host environment (19, 90). Thoughtful consideration of the population dynamics and selective pressures viruses experience is necessary before attempting mutational scanning studies *in vivo*.

One of the earliest attempts to extrapolate DMS data to natural viral evolution again comes from influenza, where DMS data were used to inform site-specific codon substitution models used in phylogenetic analysis of influenza evolution with a tool called “phydms” (126). The utility of this approach is twofold. The site-specific substitution models improve phylogenetic inference by improving model fit and estimates of evolutionary distance. The tool also identifies sites whose natural evolution diverges from empirical expectation and are, therefore, likely evolving under selective pressure from adaptive immunity or other factors missing in the laboratory setting (17, 127).

An alternative approach to phylogenetic models is the comparison of observed mutational diversity in DMS studies to the natural diversity of viral proteins. In a recent study examining the envelope protein of West Nile virus, we compared the observed site-specific preferences for residue substitutions in our DMS data to the diversity of sequences across related flaviviruses. Calculating the divergence of per-site entropy (by Kullback-Liebler divergence) in the DMS data to that in alignments of circulating viruses, we demonstrated that the DMS data were highly predictive of sequence diversity among circulating WNV viruses and that divergence increased with phylogenetic distance. Although divergence increased monotonically across more divergent mosquito-borne flaviviruses, the divergence from the DMS increased dramatically in comparison to tick-borne flaviviruses, suggesting that moving to this alternative host changed the evolutionary constraint on the E protein substantially.

Similarly, recent work has sought to compare the mutational constraints shared by evolutionary divergent HA proteins from distinct flu lineages, H3, 5, and 7, which, despite very low sequence conservation (around 40%), share highly similar structures (91). Using another measure of divergence, Jensen-Shannon divergence, the authors compared the preferences of specific substitutions at aligned sites. This analysis found preferences on the exposed surface were more similar across the highly diverged sequences, while residues in the core of the protein are more constrained likely due to the number of structural interactions in which they participate and the corresponding influence of epistasis.

Although DMS studies on related viruses are still relatively few, two recent studies reported genome-wide DMS on related EVs, EV-A (EV A71) (20) and EV-B (coxsackievirus B3) (21), enabling a proteome-wide comparison of evolutionary constraint and divergence. Our meta-analysis of these data sets revealed that, despite 50% sequence identity, the overall patterns of constraint on these viral proteomes were highly similar (120). The differences observed between the data sets highlighted potential points of structural and functional divergence, including differences in receptor usage and antibody immune pressures reflected in the capsid proteins, and differences in virus-host interactions in the non-structural proteins. Focusing on the most conserved portions of the viral proteome, we identified potential druggable sites that could be targeted in future development of pan-enteroviral drugs.

Together, these studies demonstrate the many ways DMS data can be used to inform our understanding of viral diversification in nature. Although they emphasize how structural and functional constraints are largely conserved, even across long evolutionary timescales, such comparisons can highlight regions of structural and functional divergence across related viruses. Beyond the fundamental insights into the nature of structural and functional constraint and conservation across diverse proteins, the ability to extrapolate DMS data onto natural evolution will also inform vaccine and drug design in the future.

### DMS across selective landscapes

Although often represented as fixed, evolutionary landscapes that shape viral diversity are dynamic, shifting as the immunological, cellular, and physical environments change over time and across space. As a result, the fitness of specific substitutions changes as the selective pressures shift (Fig. 2d). Applying DMS across selective gradients allows dissection of these shifts in distinct environments. A now classic, non-viral example of such a DMS study focused on ubiquitin, where fitness effects were measured under specific stress conditions (128), highlighting how changes in the cellular environment can alter the functions at specific sites. Similarly, in viral contexts, selection with specific inhibitors or in altered physical or chemical environments can provide insights into protein function and the pleiotropic effects of mutation on fitness in distinct selective environments.

A recent example is the interrogation of fitness in the EV-A71 non-structural proteins across a range of concentrations of specific virus- and host-targeted inhibitors (129). As expected, the virus-targeted inhibitors drove the enrichment of variants in their target viral proteins, while host-targeted inhibitors highlighted regions of viral proteins that bind or functionally interact with the inhibited host factors. However, beyond these expected observations, several novel findings emerged. Inhibiting the 3C protease, for example, led to mutations emerging in the other viral protease, 2A. In addition, inhibiting a host factor recruited to the replication complex by the 3A viral protein uncovered a redundant mechanism for host factor recruitment by the viral protein 2C.

A fascinating hallmark of viruses is how much can be accomplished biologically in such a relatively simple entity. This means that a given mutation will likely have effects on several functions, leading to many potential phenotypes depending on how fitness is assessed in specific selective conditions, an effect known as pleiotropy. A key element in understanding pleiotropy is determining the dimensionality of fitness and gaining a more comprehensive view of the number of environments in which a given mutation affects fitness (130). While the number of environments that one can consider can quickly exceed experimental feasibility, some work has attempted to address this framework. For example, work from yeast demonstrated that adaptive mutations can have pleiotropic effects beyond environments similar to those to which the strains were adapted (131). The ability to explicitly model these interactions provides a tractable goal that virologists should aspire to, particularly relating the fitness landscape of replication to pleiotropic effects on circumvention of countermeasures. DMS has been used to a limited extent to address pleiotropy, demonstrating proof of principle (132).

### Higher-order mutational scanning

One of the key advantages of DMS is the scale at which it can assess mutational effects. The ability to saturate a region of sequence space for a given protein or set of proteins and systematically quantify the fitness effects is powerful, but the trade-off for this breadth is limited mutational depth, usually one or two amino acid substitutions per screened variant. While much can be learned about evolutionary constraints from this approach, nature often navigates evolutionary problems via combinations of mutations. Under the most simplistic model, to understand the impact of N mutations on a given gene, one needs only to sum the N fitness effects measured in isolation. There are situations where such a model largely holds true. However, many studies have shown that the fitness effect of a given mutation can be context-dependent. The phenomenon where a mutation has different phenotypes depending on the genetic background is known as genetic epistasis (Fig. 2e). Estimates of the prevalence of epistasis vary widely between models and analytical approaches (133–135).

Considering the complexity and multifunctionality of viral proteins, it is easy to imagine that epistatic interactions are common within and between viral proteins, especially among those that interact physically and functionally to other viral proteins or host factors. Existing DMS studies have demonstrated such interactions primarily by screening libraries across diverse genetic backgrounds of a given virus strain. For example, screening an HA DMS library across different genetic backgrounds of NA demonstrated the interdependency of their fitness landscapes (136).

Epistasis also shapes the evolution and activity of single proteins, as shown in multiple screens of SARS-CoV-2 Spike. Substitutions, such as N501Y, caused large epistatic shifts in traits like binding to ACE2, leading to an expanded repertoire of antibody escape mutations (37, 68). Indeed, although DMS studies are successful in predicting the emergence of escape mutations in SARS-CoV-2, the time horizon at which these predictions are obscured by epistatic interactions is still relatively limited (65). Going forward, more must be done to directly measure high-order epistasis in viruses and understand its influence across different viral genera, especially in non-structural proteins where it has not been examined as extensively.

The primary challenge of understanding epistasis in any systematic way is the quagmire of combinatorics. As the number of residues included in a combinatorial screen increases, the number of unique variants increases exponentially (20^N^ for amino acid substitutions) to infeasible numbers. However, researchers have explored computational and experimental approaches to refine the search for epistatic interactions to libraries of tractable sizes. Analytical methods, such as statistical coupling analyses, can highlight potentially epistatically linked sites using alignments of natural diversity (137, 138). These sites can then be interrogated in targeted experimental screens. Others use the assumption that natural selection has already explored epistatic interactions and design screens based on observed mutations (33, 83). In addition, advances in oligopool engineering, such as the Spread-Out Low Diversity (SOLD) library by Twist Biosciences, provide new methods to efficiently screen combinatorial possibilities. There are still significant opportunities for both computational and experimental tools that can advance our understanding of epistatic interactions in viral proteins and overcome the challenges it poses.

### Emerging computational tools that complement DMS

To navigate the complexity of epistatic interactions and overcome the biased or available data in viral sequence databases, new computational approaches like protein language models (PLMs) are becoming essential to decipher the fundamental rules of evolutionary constraint within viral proteins. These models have the potential to use broad training data sets (that include non-viral sequences) to identify likely effects of mutation on the mutational tolerance at other sites in a protein sequence and structure (139, 140). Like much of the technology described here, these models were accelerated by the COVID-19 pandemic with the intention of identifying potential variants of concern in SARS-CoV-2 (141). A more recent study in SARS-CoV-2 highlights how these PLMs can predict effects of mutation and their effects on future mutations, potentially allowing prediction of likely evolutionary trajectories that account for epistatic interactions (142). The next frontier for these models is to better address how predicted mutational effects reflect specific phenotypes, such as protein function, transmissibility, or antigenicity, using interpretable models that clarify the reasoning behind predicted fitness changes (143).

Structural predictions, with tools, such as Alphafold and related deep learning-based approaches, have emerged as another critical tool for linking mutational effects to structure. Although these models are not strictly designed to predict the effects of point substitutions, insertions, or deletions, they can assist our interpretation in several ways. First, for sequences without experimentally determined structures, predicted structures can be useful templates for the interpretation of variant effects. In a recent study, our group used Alphafold3 and Boltz-2 predictions to interpret the effects of mutants selected under specific direct-acting and host-targeted inhibitors of EV replication (129). Mapping the fitness effects of mutation in these selected conditions onto previous unresolved structures of viral replication complex proteins, we were able to better understand the basic function of the replication machinery and interpret the mechanisms by which EVs can evade host and viral inhibition. These approaches not only allow validation of the DMS data itself, but also provides a means of validating structural predictions that are consistent with the DMS data. Formally integrating protein language and deep learning structure prediction models, therefore, represents the next logical step toward robustly predicting the structural and functional consequences of mutations across viral proteins.

## CONCLUSION AND FUTURE DIRECTIONS

Aided by rapid advances in synthetic biology and computational methods, DMS has transformed the field of viral evolution, enabling virologists to connect viral genotype to phenotype at a proteome-wide scale. Looking ahead, the field is poised to address several key challenges. Future work will focus on the expanding screens to include broader environmental conditions, enabling better extrapolation of *in vitro* DMS data to *in vivo* contexts. As the number and scale of DMS studies expand, it will improve our ability to look deeper into the evolutionary past, with more comparative studies across viral families, and future through systematic exploration of higher-order genetic interactions (epistasis). The synergies between DMS and emerging computational tools, such as protein language models and advanced structural prediction, will enhance our ability to interpret the mechanisms underlying mutational phenotypes. Through all of these advances, DMS will continue to refine our basic understanding of viral molecular biology while enhancing its translational impacts in anticipating viral emergence and designing more robust vaccines and antivirals.

All these efforts and conceptual advances will be accelerated by collaborative communities of researchers performing and analyzing DMS work in virology. A notable example is the recent establishment of the Virology Interest Group by members of the Atlas of Variant Effects (AVE) (144), a broad community of researchers working on methods to measure, quantify, and predict the effects of variants and develop minimal reporting standards for such studies. The establishment of the Virology Interest Group within AVE brings together experimental and computational researchers to discuss how to address analytical challenges, develop new experimental paradigms, and expand the use of mutational scanning to new problems in virology.
